# Suicide risk after psychiatric discharge: study protocol of a naturalistic, long-term, prospective observational study

**DOI:** 10.1186/s40814-020-00685-z

**Published:** 2020-09-30

**Authors:** Tim J. Krause, Annette Lederer, Magdalena Sauer, Jasmin Schneider, Cathrin Sauer, Burkhard Jabs, Elmar Etzersdorfer, Axel Genz, Michael Bauer, Susann Richter, Dan Rujescu, Ute Lewitzka

**Affiliations:** 1 Department of Psychiatry, Psychotherapy and Psychosomatic Medicine, University Hospital Halle, Faculty of Medicine, Martin-Luther-Universität Halle-Wittenberg, Dresden, Germany; 2 Department of Psychiatry and Psychotherapy, Municipal Hospital Dresden, Dresden, Germany; 3 Department of Psychiatry and Psychotherapy, Furtbach Hospital, Stuttgart, Germany; 4 Department of Psychiatry and Psychotherapy, University Hospital Magdeburg, Faculty of Medicine, Otto-von-Guericke-Universität Magdeburg, Magdeburg, Germany; 5 Department of Psychiatry and Psychotherapy, University Hospital Carl Gustav Carus, Technische Universität Dresden, Fetscherstr. 74, D-01307 Dresden, Germany

**Keywords:** Suicide risk, Suicidality, Suicidality after discharge, Suicide prevention, Affective disorders, Schizophrenia, Perfectionism, Public health

## Abstract

**Background:**

Suicide risk of psychiatric patients has proven to be strongly increased in the months after discharge from a psychiatric hospital. Despite this high risk, there is a lack of systematic research on the causes of this elevated suicide risk as well as a lack of treatment and intervention for patients at high risk after discharge. The main objective of this pilot study is, firstly, to examine the factors contributing to the elevated *suicide risk* and, secondly, to investigate whether an additional setting of care starting at discharge may reduce *suicidality.*

**Methods:**

In this multi-centre pilot study, treatment as usual is complemented by an additional 18-month post-discharge setting of care for psychiatric patients at high risk for suicide. Two groups of patients differing in the amount of post-discharge personal contacts will be compared. One group of patients will be offered continuous personal contacts after discharge (months 1–6: monthly contacts; months 6–18: every 2 months) while another group of patients will receive contacts only at months 6, 12, and 18 after discharge. Data on suicidality, as well as associated with other symptoms, treatment, and significant events, will be collected. In the case of health-related severe events, the setting of care allows the patient to have the opportunity to connect with the doctor or therapist treating the patient.

**Discussion:**

The results of this study will contribute to identifying critical factors raising suicide risk after discharge and will demonstrate the potential influence on suicide prevention of a setting of care with regular personal contact after discharge.

**Trial registration:**

ZMVI1-2517FSB135 – funded by the German Federal Ministry of Health.

## Background

Approximately 800,000 people die by suicide globally every year [1]. According to the World Health Organization (WHO), the average suicide rate is 10.6 suicides per 100,000 persons in 2016 [2]. Europe’s average suicide rate is 15.4; the highest rate of all world regions.

Several programmes of the European Union focussed on reducing suicide rates. In Germany, a national suicide prevention programme (Nationales Suizidpräventionsprogramm, NaSPro) was initiated in 2002 by the German Association for Suicide Prevention (Deutsche Gesellschaft für Suizidprävention, DGS), and various activities were conducted.

To understand the heterogeneous and complex phenomenon of suicide, psychological autopsy studies have become a very valuable tool in past decades [3]. Up to 90% of people that died by suicide suffered from mental disorders. A systematic review of the literature showed proportions of mental disorders of 91% in 54 case series and 90% in 22 case-control studies [4]. A review of 27 psychological autopsy studies revealed a rate of 87.3% of all 3275 suicides that were diagnosed with a mental disorder prior to their death [5].

Periods of psychiatric inpatient treatment are considered as particularly vulnerable for suicide since hospitalisation only becomes necessary when symptomatology reaches a critical point, like patients who might endanger themselves or others. Suicide in hospitals was an object of investigation in various studies [6, 7]. Suicide risk is not the same across all treatment phases. The highest risk was measured during the months following a hospital stay. Though only a few data exist, they show remarkably similar results despite all differences in health care systems [8–13].

Investigations in Germany strongly revealed the highest risks of suicide for patients with schizophrenia and depression in the first months after discharge. In the 2 years following inpatient treatment, the risk to die by suicide was increased by a 66-time fold for male patients with schizophrenia, 110-time fold for female patients with schizophrenia, 111-time fold for male patients with depression, and a 41-time fold for female patients with depression [8].

A population-based study in the Oxford health region, UK, calculated the risk of suicide within 1 year after psychiatric discharge. Suicide rates were measured per 1000 person-years at risk and the standardised mortality ratio (SMR) for suicide. The SMR is a ratio of the observed number of deaths in a study group, and the number of deaths would be expected. The value among the general population was 1. For male patients, the SMR to die by suicide in the first 28 days after discharge was 213 (95% CI 137–317); the SMR for female patients was 134 (67–240). The suicide rate in the first 28 days after discharge was 7.1 (4.1–12) times higher for male patients and 3.0 (1.5–6.0) times higher for female patients than the level during the remaining 48 weeks of the first year after discharge [9].

A further study investigated suicide rates within a year of discharge from psychiatric inpatient care in Hong Kong. Discharges from all psychiatric hospitals or psychiatric wards in general hospitals from 1997 to 1999 were followed up for suicides and undetermined causes of deaths by record linkage with the Coroner’s Court. Suicide rates per 1000 person-years at risk and SMRs were calculated. In this study, 21,921 patients with an age of over 15 years were discharged from psychiatric hospitals from 1997 to 1999. Overall, 280 patients died by suicide within 1 year; 85 suicides (30%) occurred within 28 days after discharge. The SMRs for suicide in the first 28 days after discharge were 113 (95% CI = 86–147) for males and 178 (95% CI = 132–235) for females. These rates were 4.6 (95% CI = 3.2 to 6.3) times higher for males and 4.0 (95% CI = 2.7 to 5.6) times higher for females compared to the rate in the rest of the year [10]. A Korean study investigating suicide rates in the year following psychiatric discharge found that for all patients admitted to Seoul hospitals in Korea for psychiatric disorders between 1989 and 2006 (*N*, 8403), SMRs in the year following discharge were 49.7 for males and 45.5 for females [11].

A systematic review and meta-analysis on suicide rates after discharge examined publications from 1946 to 2016. Altogether, 100 studies were analysed that included 17,857 suicides. The pooled suicide rate was 484 suicides per 100,000 person-years (95% CI = 422–555). The suicide rate was highest within 3 months after discharge (1132; 95% CI = 874–1467). In the period from 3 months to 1 year, suicide rates were 654, 494 in the period after year 1 to year 5 and 366 for the follow-up period of 5 to 10 years, and 277 for studies with follow-up periods longer than 10 years [12].

A review on suicide rates following hospital discharge examined 48 studies from 1964 to 2017. The pooled annualised suicide rate was 241 (95% CI = 238–243) per 100,000 person-years including 41 studies [13].

Several causes of this excessively increased suicide risk in the months after discharge may be considered as follows:

- Patients were not treated successfully. There was treatment failure or only a partial response to treatment.
- New or different problems developed after discharge that overburdened the individuals’ potentially reduced coping strategies.
- Because of other reasons, clinical symptoms worsened, for example, due to a lack of adherence to medication.
- The influence of the therapeutic relationship—usually ending with discharge. To date, there have been only a few studies and statistically reliable results. One study analysing German railway suicides showed an odds ratio of 22.86 for the change of therapist during the hospital stay and the association with suicide [14].
- Other causes such as disappointment and hopelessness after failing the highest level of care, the associated stigma of being admitted to a mental health facility, being in contact with patients that are potentially very sick, and learning about other more lethal methods of suicide.

The group of patients being discharged from a psychiatric inpatient stay represents a population at risk for suicide that does not receive specialised care regardless of the type of mental disorder. Specific care concepts focussing on these patients with tremendously increased suicide risk rarely exist.

Moreover, systematic investigations on the complex and heterogeneous background of increased suicide mortality after discharge from psychiatric hospitals and the influences of different treatment settings on suicide prevention were not carried out.

For this reason, the presented study examines the following questions:

i) What might influence the increased suicide mortality in the months after discharge from an inpatient psychiatric hospital stay? Which factors might influence suicide mortality?
ii) Is it possible to reduce suicidality after discharge by establishing a specific setting of care?

This project is of considerable socio-political interest. If it is possible to reduce suicide rates or suicidal behaviour by implementing a specific setting of care, this concept might be transferred to other mental health systems.

## Methods/design

The objective of this multi-centre trial is, firstly, to identify factors affecting suicide risk after psychiatric discharge and, secondly, to investigate whether a specific setting of care defined by regular personal contacts influences the elevated suicide risk after discharge from a psychiatric hospital.

This naturalistic study will allocate patients with schizophrenia, schizotypal and delusional disorders (ICD-10: F2), or mood disorders (ICD-10: F3) [15] as principal diagnosis either to a group with only a single contact 6, 12, and 18 months after discharge or to a group where personal contacts are organised monthly to every 2 months in the 1.5 years following discharge. These study contacts are an addition to treatment as usual for all patients and do not include medical or therapeutic treatment. Following discharge, the protocol will continue even if the patient gets admitted to the hospital again. By-phone visits will be allowed as alternatives.

The hypothesis to be tested is whether a specific setting of care reduces the suicide risk and does this particular setting of care plus regular treatment as usual lead to a significant reduction of suicidality compared to a less intense setting of care plus regular treatment as usual in the months following a psychiatric hospital stay.

The primary endpoint of the study is the evaluation of suicidality. Suicidality is measured by the score of the clinician-administered Sheehan Suicidality Tracking Scale (S-STS, [16]) at 6, 12, and 18 months after discharge.

Secondary endpoints include the score of the patient self-reported S-STS, changes in depressive symptoms as indicated by the Montgomery Asberg Depression Rating Scale (MADRS, [17]), changes in psychotic symptoms as indicated by the Brief Psychiatric Rating Scale (BPRS, [18]), and changes in the Visual Analogue Mood Scale (VAMS, [19]) at 6, 12, and 18 months after discharge. Due to the possibility of contacting the doctor/therapist and/or a close person of the patient in case of non-appearance to an appointment combined with failure, to reach the patient, we want to gain information about the state of health and ascertain possible suicides. At every study visit, it is asked for suicide attempts.

The number of days of psychiatric hospital treatment, as well as quantity and dosage of medication and the number of psychotherapy sessions, will be recorded. Furthermore, critical stress-related events after discharge and the subjective degree of stress will be collected.

Basic descriptive data on regional health care services/differences at the four participating hospitals will be collected (e.g., number of patients presenting with disorders of F2 and F3-section (ICD-10), regional characteristics, differences in networks of psychiatric support, and treatment after discharge). Recruitment started in 2018 and will end in 2021.

### Study population and eligibility

Patients will be recruited from the four participating psychiatric hospitals in Germany (Department of Psychiatry and Psychotherapy, University Hospital Carl Gustav Carus, Dresden; Municipal Hospital Dresden, Department of Psychiatry and Psychotherapy, Dresden; Department of Psychiatry, Psychotherapy and Psychosomatics University Hospital Halle; Department of Psychiatry and Psychotherapy Furtbach Hospital, Stuttgart). All patients meeting the inclusion criteria will be informed about the study. Inclusion criteria are 18 years of age and older, admitted as an inpatient, presenting with either a mood/affective disorder (F3 section of ICD-10) or schizophrenia, schizotypal or delusional disorder (F2 section of ICD-10) and written informed consent. Patients agreeing to participate in the study have a choice to which group they want to belong to. A randomised allocation is not made for ethical reasons. Patients with legal guardians are excluded from the study. Patients are not given any incentive or compensation for participation.

### Course

All patients receive a baseline visit (V0) during the week prior to discharge. At the baseline visit, data on demographics, substance use, psychiatric and medical history and treatment, history of suicidality, and family history of suicidality are documented.

**Fig. 1:**
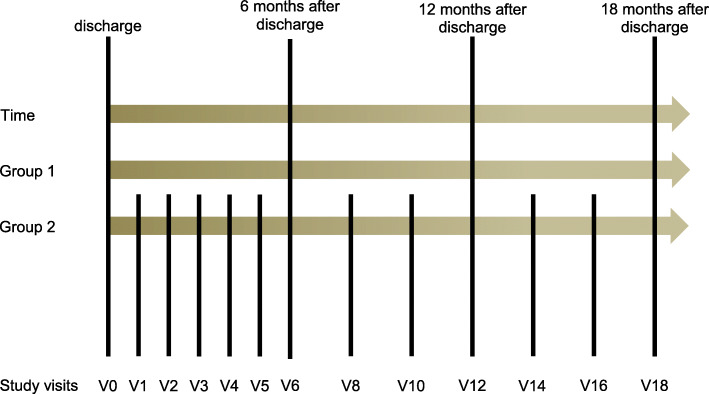
Trial flow

In group 1, study contacts are scheduled at 6 (V6), at 12 (V12), and at 18 (V18) months after discharge from the psychiatric hospital. In group 2, more frequent study contacts are organised: During a period of 6 months after discharge, study visits are scheduled monthly (V1–V6), and subsequently every 2 months during the next period of 6 to 18 months after discharge (V8–V18). There will be a total of 3 study visits in group 1 and 12 study visits in group 2. The protocol (Fig. 1) accepts flexibility in terms of time, plus/minus 1 week, for study visits in the first 6 months after discharge, as well as a plus/minus 2 weeks tolerance for study visits V8 to V18. In case a patient gets admitted to a hospital while participating in the study, the course will be continued as conducted. Study visits include an evaluation of events that occurred between the study visits, e.g., stress-related events, inability to work, days of inpatient treatment, change or discontinuation of any treatment, and change of doctors, or therapists. Both baseline and study visit additionally includes several assessments recording symptomatology described in the next section.

Both groups will receive treatment as usual in terms of regular meetings with their general practitioner, psychiatrist, and/or psychotherapist. The idea of the present study is to create a four eyes principle. The study setting includes an option to connect with the patients’ doctors or therapists as well as a person the patient chose in advance. It applies in case of health-related severe events (e.g., increasing suicide risk, suicide attempt). It enables the investigator to provide support, e.g., by coordinating further procedures with the patients’ psychiatrist/psychotherapist (provided that the patient voted for this option in written consent). If confronted with acute suicide risk, the investigator may also decide to follow the hospital’s emergency procedure in case of risk of self-harm and immediately present the participant to a psychiatrist on site. Connecting with doctors and therapists or a close person will also be possible in case of non-appearance to an appointment combined with a failure to reach the patient. This also allows us to gain important information on a potential suicide risk or suicide*.*

### Assessments

#### Clinician-administered scales

Sheehan Suicidality Tracking Scale (S-STS, [20]) is used to assess suicidal thoughts and/or behaviour, both clinician and self-reported. Items of the S-STS are scored on a 5-point Likert scale (0 = not at all, 1 = a little, 2 = moderately, 3 = very, and 4 = extremely). The Montgomery Asberg Depression Rating Scale (MADRS, [16]) will assess depressive symptoms; the Brief Psychiatric Rating Scale (BPRS, [17]) assesses psychotic symptoms. Investigators will also make use of the AMDP-rating scale to evaluate clinical psychopathology (Arbeitsgemeinschaft für Methodik und Dokumentation in der Psychiatrie, AMDP [19]). A rater training, as well as an evaluation of inter-rater reliability, was held at the beginning of the study to minimise differences between investigators and centres.

#### Patient-administered scales

The self-rating version of S-STS will be used as a self-rating instrument on suicidality. The Visual Analogue Mood Scale (VAMS) [18] will be used to reflect the current subjective mental state. Both will be applied at baseline contact and at each following contact in both groups. At the baseline visit only (V0), patients of both groups will be asked to complete a questionnaire on perfectionism [21–24], as well as the FPI-R Questionnaire (Freiburger Persönlichkeitsinventar, [25]) to investigate whether and to what extent various psychological constructs (e.g., perfectionism, aggressiveness) are associated with suicidality. Additionally, we ask patients to fill out the Resilience Scale (RS13) [26] to investigate possible influences on the development of suicidality. The RS13 measures resilience on a 7-point scale and is the short German version of the RS-25 from Wagnild and Young [27].

### Power analysis and sample size calculation

The presented multi-centre trial is designed as a pilot study. We, therefore, did not conduct a statistical power analysis or sample size calculation. Yet, we estimate a minimum of 300 patients treated with ICD-10 F2- or F3-diagnosis in each study centre every year. Based on clinical experience, we strive for at least 100 patients per study centre.

### Statistical analysis

Descriptive statistics covering sociodemographic and clinical baseline data will be reported using proportions, means, and standard deviations as well as Chi-square tests, *t* tests, and Mann-Whitney tests as appropriate. Results will be presented with 95% confidence intervals where applicable. Proportions of adverse events will be summarised. Sociodemographic and clinical variables that differ significantly between groups at baseline will be included as covariates in further analyses. To identify potential explanatory variables on suicidality in the weeks and months after discharge, a potential correlation of variables will be assessed by frequencies, percentages, means and standard deviations, correlation coefficients, etc. Variables that are identified as potentially influencing suicidality will be included as factors in further analyses.

Normal distribution will be assessed by Shapiro-Wilk tests and by visual inspection of histograms. Homogeneity of variances will be assessed using Levene’s test.

Statistical tests will be two-sided using an alpha level of 0.05. All analyses will be conducted using IBM SPSS Statistics for Windows, version 25.

#### Primary outcome measures

Group comparison of suicidality measured by the score of the clinician-administered Sheehan Suicidality Tracking Scale will be conducted using analysis of covariance (ANCOVA). Baseline variables that differed significantly between groups, as well as potentially influencing risk factors, will be included in the analysis.

#### Secondary outcome measures

Group comparisons of secondary outcome measures will be conducted using analysis of (co)variance (AN(C)OVA), Poisson regression, logistic regression, *t* tests, Chi-square tests, or non-parametric methods as appropriate. The course of suicidality measured with S-STS, as well as scores of the other scales, will be analysed within groups using repeated measures AN(C)OVA for both groups separately comparing baseline values with scores at 6, 12, and 18 months.

### Data and safety management

The study protocol conforms to GCP (Good Clinical Practice) standard procedures, and all investigators participated in GCP courses. Monitor visits are carried out by the staff of the University Hospital Carl Gustav Carus. Study data will be stored for 10 years and will be destroyed afterwards. As per protocol, all serious adverse events will be documented and sent to the sponsor. Very experienced and trained investigators perform the assessments. They took part in mandatory interrater training.

### Ethical considerations

Research on suicide has to consider specific requirements and demands concerning study protocols, study designs, and methodological concepts. All psychiatric patients participating in this study undergo regular available treatment (treatment as usual, TAU). While participation in any of both study groups includes additional contacts to study investigators, a four eyes principle is introduced to meet the particular requirements of care psychiatric patients might need after discharge from the hospital. Participants of both groups agree that in case of significant events (e.g., suicide attempt, suicide), the study investigator is allowed to contact the person the patient chose in advance. The Ethics Committees have approved the study.

## Discussion

As suicide risk in psychiatric patients is highly increased in the period after discharge from a psychiatric hospital, there is a high need to understand the complex phenomenon of suicidality after discharge. To date, however, this high-risk group for suicide has rarely been approached by research. Considering this, the proposed multi-centre pilot study will collect various and heterogeneous data on patients after discharge from psychiatric hospitals, while at the same time offering patients a personal contact setting in the period following hospital treatment, which includes the option to connect with a doctor or therapist. The results of this pilot study should contribute to identifying factors that raise suicide risk after discharge as well as indicate whether a contact setting may be a promising prospect in terms of suicide prevention.

The approach of this study aims at being in accordance with clinical reality, which is essential for a possible future transformation of our results into everyday clinical practice. Being a multi-centre study, the trial population is more heterogeneous, meaning more information is expected to be gained, and more patients can be included.

Several other aspects may play a role within this selected care approach. Study visits will occur in addition to regular care, which means one more social contact independent of the content, which might be especially relevant for isolated patients. Moreover, this increases the chance of recognising suicidal tendencies and prevents them (4 eyes principle). The study appointments establish a link between the end of inpatient treatment and further outpatient care. Some patients may feel less of a break in their relationship due to the familiar environment. Another difference is that the study visits are conducted as safety-focused contacts, which also means that it is possible to have more time for the patient than a regular medical contact would be able to offer. In the case of elevated suicide risk, it is possible to discuss the following procedure within an interdisciplinary team, either within the hospital but also within the study team. Within the study visit, a semi-standardised conversation flow is used. The patients can better adjust to appointments and reflect on their development. There also may be more openness in the conversation. The expectations of the study staff are different from regular doctor or therapist appointments.

The present study has several limitations. First, patients with a principal diagnosis of only categories F2 and F3 of ICD-10 (schizophrenia, schizotypal or delusional disorders, depression, bipolar disorder) will be included. In contrast, all other diagnoses will be accepted as comorbidities only. This procedure leaves out several groups of patients. We tried to restrict the heterogeneity of our study population in favour of receiving insights that are more meaningful for a well-defined group of patients at this first step of approaching very critical aspects of suicidality. Second, a bias may arise from the fact that patients have a choice of which group they would like to be placed in, either group 1 or group 2. On the other hand, this choice can offer useful insights into real-world clinical aspects by reflecting patients’ different characteristics as well as needs and motivation to make use of suicide prevention measures providing personal contact. This is in line with the ethical requirements of not withholding any condition from participants.

As a limitation of our study, we do not examine developments during the first days after discharge. The first study contact is scheduled 1 month after discharge. A recently published meta-analysis comprising 34 publications showed a pooled estimate of the suicide rate of 2950 suicides per 100,000 person-years (95% CI = 1740–5000) [28]. The pooled estimate of the suicide rate during the first month after discharge was 2060 per 100,000 person-years (95% CI = 1300–3280).

## Conclusions

The proposed pilot study will provide insights into the causes of the highly elevated risk of suicide in the period after discharge from a psychiatric hospital. It may point out further research questions and crucial features of suicide prevention procedures.

## Data Availability

Not applicable yet

## Abbreviations

WHO: World Health Organization
NaSPro: Nationales Suizidpräventionsgprogamm (National Suicide Prevention Program)
DGS: Deutsche Gesellschaft für Suizidprävention (German Association for Suicide Prevention)
UK: United Kingdom
SMR: Standardised mortality ratio
CI: Confidence interval
ICD: International Classification of Diseases
S-STS: Sheehan Suicidality Tracking Scale
MADRS: Montgomery Asberg Depression Scale
BPRS: Brief Psychiatry Rating Scale
VAMS: Visual Analogue Mood Scale
AMDP: Arbeitsgemeinschaft für Methodik und Dokumentation in der Psychiatrie
FPI-R: Freiburger Persönlichkeitsinventar
RS13: Resilience Scale
IBM: International Business Machines Corporation
SPPS: Statistical Package for the Social Sciences
ANCOVA: Analysis of covariance
GCP: Good Clinical Practice
TAU: Treatment as usual
